# Atypical Cutaneous Manifestation Leading to the Diagnosis of Chronic Lymphocytic Leukemia

**DOI:** 10.7759/cureus.110370

**Published:** 2026-06-06

**Authors:** Muhammad Memon

**Affiliations:** 1 Internal Medicine, Christiana Care Health System, Newark, USA

**Keywords:** chronic lymphocytic leukemia, clinical hematology, dermatology, diffuse rash, hematology-oncology, skin disease

## Abstract

We report a 64-year-old male with rheumatoid arthritis and Raynaud’s phenomenon who presented with diffuse arthralgias and progressive ulcerating lesions involving the palms and elbows. Differential diagnoses included recurrent Rickettsial infection, vasculitis, pyoderma gangrenosum, and cutaneous tuberculosis. Laboratory studies demonstrated marked leukocytosis (135.4 ×10⁹/L), while computed tomography (CT) imaging revealed hepatomegaly, lymphadenopathy, and pulmonary micronodules concerning for infiltrative disease. Additional history revealed a 40-pound unintentional weight loss over three months. Peripheral flow cytometry established a new diagnosis of chronic lymphocytic leukemia (CLL), and skin punch biopsy demonstrated epidermal necrosis with dense dermal lymphoid infiltrates consistent with leukemia cutis. This case highlights leukemia cutis as a rare initial manifestation of CLL that may mimic infectious or autoimmune conditions, emphasizing the importance of thorough evaluation of unexplained cutaneous lesions and significant leukocytosis to facilitate earlier diagnosis and treatment consideration.

## Introduction

Chronic lymphocytic leukemia (CLL) is a hematologic malignancy characterized by the clonal proliferation of mature but functionally incompetent B lymphocytes [1]. CLL is the most commonly diagnosed leukemia in the Western world, accounting for 30% of all leukemias [1]. It is usually diagnosed through routine blood work or through typical symptomatic findings of fatigue, weight loss, night sweats, lymphadenopathy, and splenomegaly, but it is rarely first seen with the initial presentation of skin manifestations [2]. Leukemia cutis is a rare skin presentation of CLL that often involves the face and manifests as macules, papules, plaques, nodules, ulcers, or blisters.

## Case presentation

A 64-year-old male with past medical history significant for rheumatoid arthritis not on disease-modifying antirheumatic drugs (DMARDs), Raynaud's phenomenon, presented to the hospital with body-wide joint aches and four days of raised skin lesions on his bilateral palms that gradually ulcerated and spread to his elbows. He also noticed worsening of his chronic joint pain during this time. He was recently diagnosed with Rocky Mountain spotted fever a year ago, with a similar presentation, and was treated with a 60-day course of doxycycline. At that time, the patient had leukocytosis, shown in Table 1, with a lymphocytic predominance.

**Table 1:** Complete blood count noting marked leukocytosis compared to prior admission result. WBC: white blood cell, Hgb: hemoglobin

	Patient value	Reference range
WBC (prior to admission)	24 (x10^3^/uL)	4.5-11.0 (x10^3^/uL)
WBC (this admission)	135.4 (x10^3^/uL)	4.5-11.0 (x10^3^/uL)
Hgb (this admission)	9.8 g/dL	13.3-17.7 g/dL
Platelets (this admission)	161 (x10^3^/uL)	150-400 (x10^3^/uL)

Skin lesions ranged in presentation from nodules to large, erythematous macular lesions with evidence of central necrosis. Initial concern was for possible recurrent Rickettsial disease with previous positive titers, pyoderma gangrenosum, and/or vasculitides or cutaneous TB with a history of incarceration. Complete blood count (CBC) showed leukocytosis with no atypical lymphocytes noted or bandemia, shown in Table 1.

**Figure 1:**
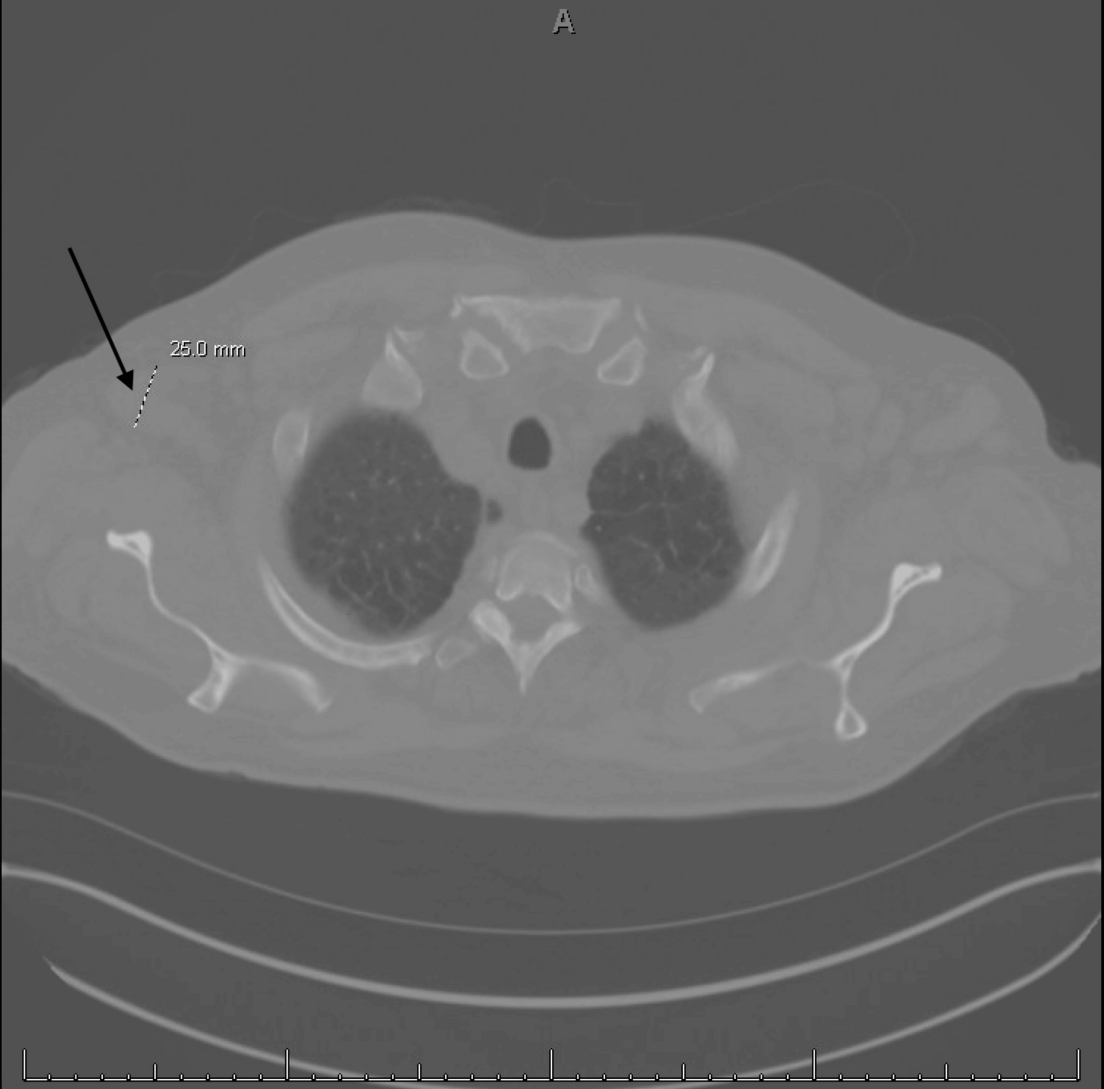
Enlarged axillary lymph node measuring 2.5 cm.

**Figure 2:**
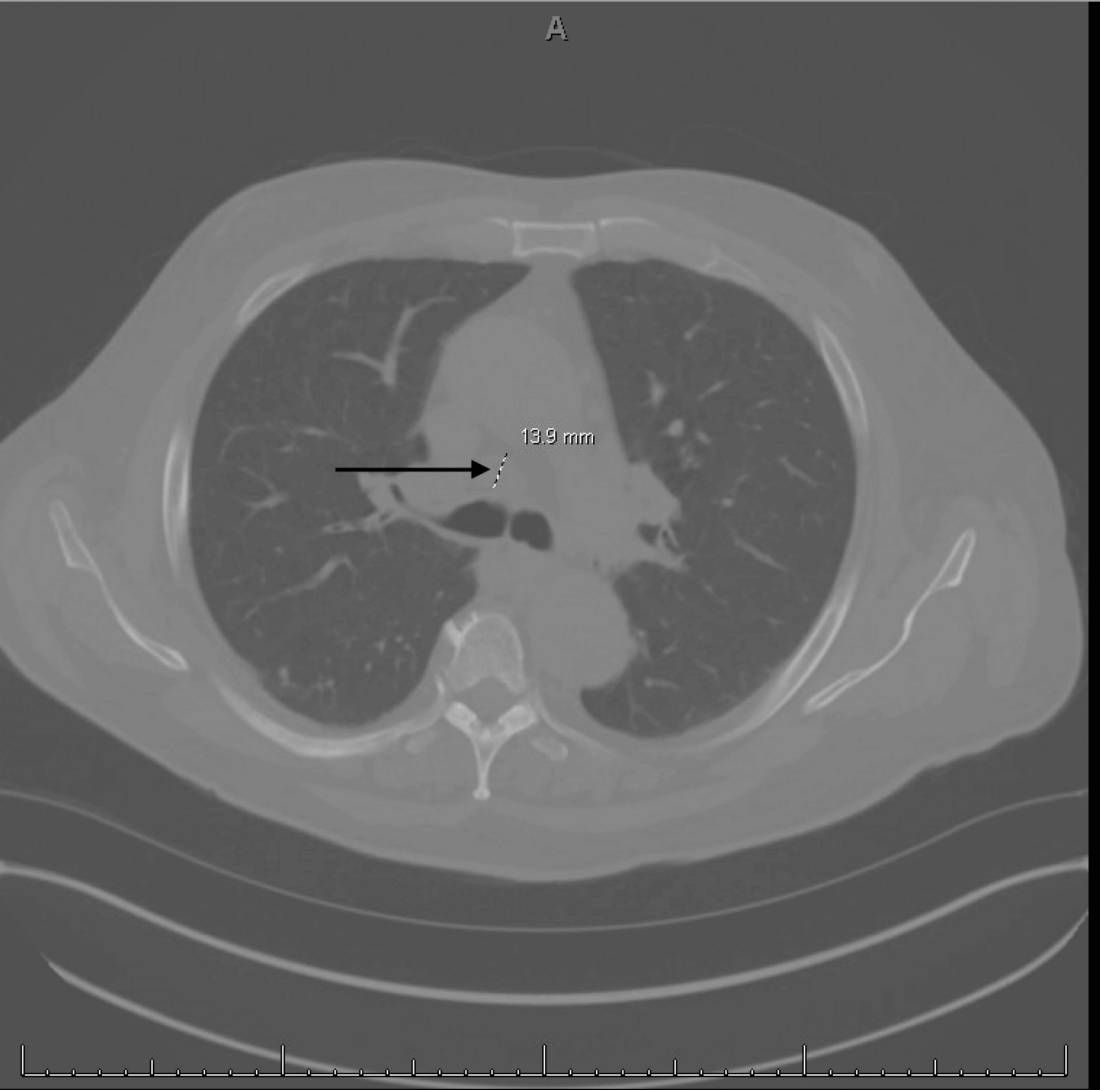
Enlarged para-aortic lymph node measuring 1.3 cm.

**Figure 3:**
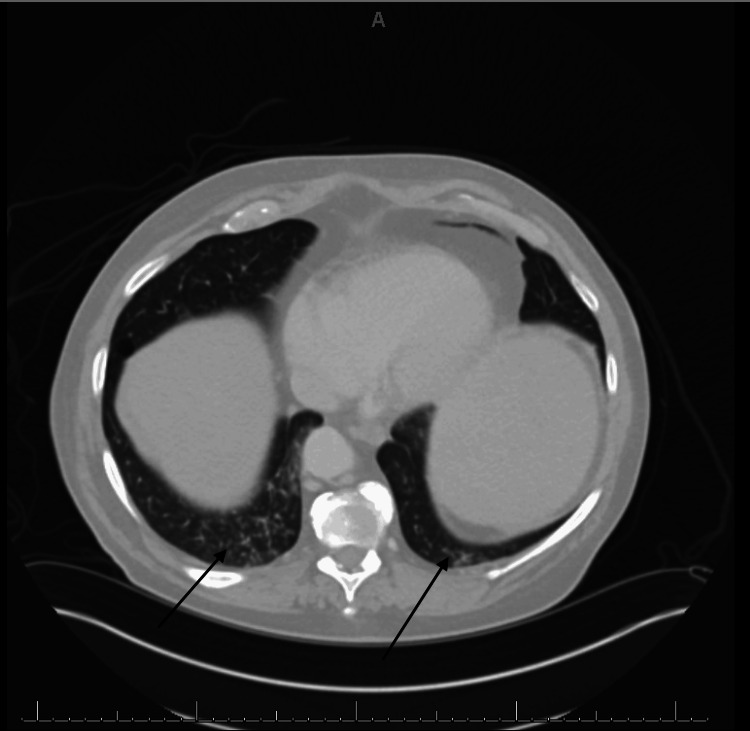
Numerous lung micro-nodules seen in the bases.

**Figure 4:**
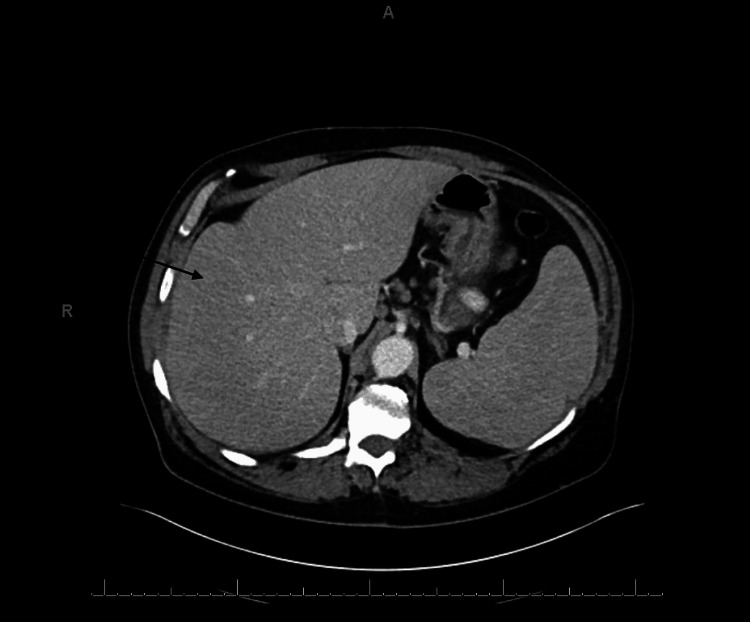
Marked hepatosplenomegaly.

Computed tomography (CT) showed hepatomegaly with evidence of portal hypertension, retroperitoneal and pelvic lymphadenopathy, and numerous micronodules in the lung bases consistent with systemic disease and infiltrative neoplasia, as shown in Figures 1-4.

**Figure 5:**
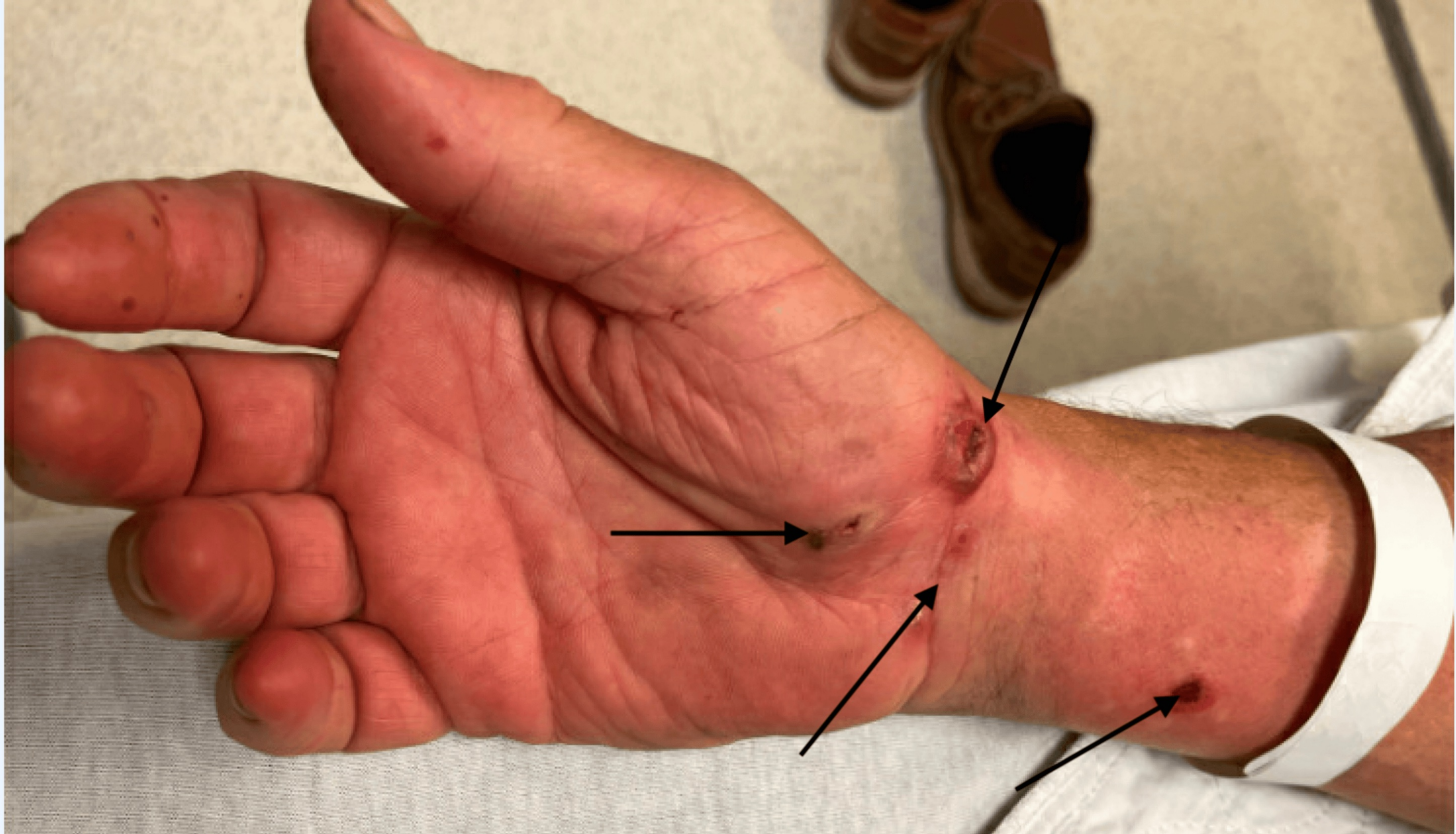
Rash on the right forearm.

**Figure 6:**
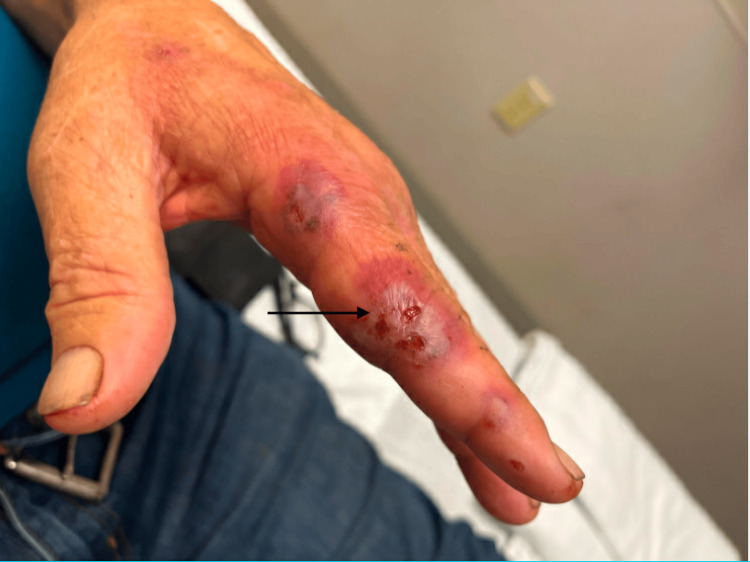
Rash on the index finger of the left hand.

**Figure 7:**
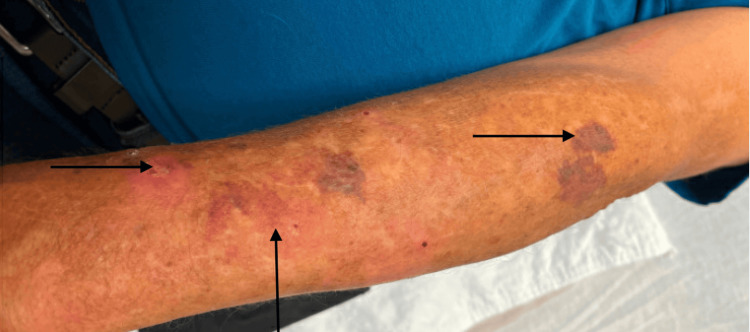
Rash on the left arm.

Further history revealed a 40-pound weight loss over three months. The patient was initially started on doxycycline for concern of recurrent Rickettsial infection, with repeat titers and other tick-borne illness testing ordered, although concern for malignancy was high. Peripheral flow was performed results indicated a new diagnosis of CLL, with a punch biopsy of the skin lesion performed and showing epidermal necrosis with a dense dermal lymphoid infiltrate, consistent with CLL. The patient was medically stable for discharge and opted to continue his care outpatient. The raised skin lesions are seen in Figures 5-7.

## Discussion

This case illustrates the potential of CLL to present initially with leukemia cutis, with the value of a complete history and physical. Leukemia cutis can present as low as 4% of total CLL cases and therefore can be a rare but missed initial finding of the disease [3]. Although it is unknown if this patient's initial presentation of Rickettsial infection was a “red herring” and he presented with leukemia cutis at his prior presentation, it is rare for the cutaneous manifestation to resolve spontaneously, although there are some case reports where this is mentioned [4]. Cutaneous manifestations of CLL may mimic other conditions, including Rickettsial infection, inflammatory dermatoses, or vasculitis, particularly in patients with underlying autoimmune disorders, such as rheumatoid arthritis [5].

Although the exact mechanism of cutaneous manifestations is not known, we believe that cutaneous leucocyte-associated antigen (CLA) receptor and CC chemokine receptor 4 (CCR4) on dysfunctional leukemic cells interact with dermal-specific receptors (E-selectin) that help migrate and bind leukemic cells into the dermis [5]. While it is an uncommon manifestation, early recognition of the presentation with correlation with patient history and exam findings can help diagnose patients with CLL earlier. The survival rates of patients with CLL are not affected by leukemia cutis [6]. Usually, asymptomatic or early CLL can be observed and does not require immediate treatment [1]. However, with our patient who seems to have rapid progression and cutaneous manifestations, treatment should be initiated. CLL can be treated depending on genetic risk factors that can determine what agents to use [1]. With second-generation covalent BTK inhibitors versus venetoclax plus obinutuzumab, depending on genetic testing. Leukemia cutis is managed with standard systemic CLL therapy, and the cutaneous manifestations should resolve. Radiotherapy to the area is reserved for refractory skin involvement [1].

## Conclusions

This case highlights leukemia cutis as a rare initial presentation of CLL and underscores the importance of maintaining a broad differential diagnosis in patients presenting with unexplained cutaneous lesions, constitutional symptoms, and marked leukocytosis. Cutaneous manifestations of CLL may mimic infectious, autoimmune, or inflammatory conditions, potentially delaying diagnosis. Early recognition of atypical presentations with timely hematologic evaluation and biopsy can facilitate earlier diagnosis, risk stratification, and initiation of appropriate treatment in patients with progressive disease features.
